# A Pilot Evaluation of a Tutorial to Teach Clients and Clinicians About Gambling Game Design

**DOI:** 10.1007/s11469-017-9816-1

**Published:** 2017-11-09

**Authors:** Nigel E. Turner, Janine Robinson, Kevin Harrigan, Peter Ferentzy, Farah Jindani

**Affiliations:** 1 Institute for Mental Health Policy Research, Centre for Addiction and Mental Health, 33 Russell Street, Toronto, M5S 2S1 Canada; 2 Problem Gambling Institute of Ontario, Centre for Addiction and Mental Health, Toronto, Canada; 3 Dalla Lana School of Public Health, University of Toronto, Toronto, Canada; 4 Gambling Research Lab, University of Waterloo, Waterloo, Canada; 5 Education and Training, Centre for Addiction and Mental Health, Toronto, Canada

**Keywords:** Problem gambling, Tutorial, Electronic gambling machines, Prevention, Treatment

## Abstract

This paper describes the pilot evaluation of an Internet-based intervention, designed to teach counselors and problem gamblers about how electronic gambling machines (EGMs) work. This study evaluated the tutorial using assessment tools, such as rating scales and test of knowledge about EGMs and random chance. The study results are based on a number of samples, including problem gambling counselors (*n* = 25) and problem gamblers (*n* = 26). The interactive tutorial was positively rated by both clients and counselors. In addition, we found a significant improvement in scores on a content test about EGM games for both clients and counselors. An analysis of the specific items suggests that the effects of the tutorial were mainly on those items that were most directly related to the content of the tutorial and did not always generalize to other items. This tutorial is available for use with clients and for education counselors. The data also suggest that the tutorial is equally effective in group settings and in individual settings. These results are promising and illustrate that the tool can be used to teach counselors and clients about game design. Furthermore, research is needed to evaluate its impact on gambling behavior.

Empirical studies have found that gambling machines are associated with more gambling related problems than other forms of gambling (Breen and Zimmerman 2002; Dorion and Nicki 2001; Griffiths 1993; MacLaren 2016; Schull 2005, Schüll 2012; Urbanoski and Rush 2006). Exactly why this is the case is still debated (Dowling et al. 2004; Mizerski et al. 2002). In addition, according to Williams and Volberg (2013), problem gamblers (combining “pathological” gamblers and subclinical problem gamblers) account for 31.2% of the expenditures on EGMs in Ontario (p. 46). To complicate matters, there are several different types of EGMs—including spinning reel slot machines, video slot machines, video poker, video keno, and multigame machines—and the features of these machines, not to mention the variety of gambling machines, are continuously increasing (G2E 2009; IGT 2009; Turner and Horbay 2004; Turner 2011a). Few features are common to all types of EGMs. Our approach to this issue was to develop a tutorial to educate gamblers about how EGMs work. The hypothesis is that an educated player will be less likely to develop a problem. Furthermore, the tutorial might assist problem gamblers in recovery and relapse prevention. To accomplish this goal, we developed an online tutorial on how EGMs work that teaches the users the principles underlying vital aspects of EGMs, including how volatility hides the house edge, the difference between the short-term experience of playing and the long-term outcome, false wins, the futility of chasing, the continuously running nature of the random number generator (RNG) in EGMs, and the cost of play. This paper reports on the results of a pilot evaluation of the tutorial.

It is well known that many gamblers lack a real understanding of the nature of random chance, probability, and the house edge. Research has shown that persons with problem gambling have a poorer understanding of the nature of random chance compared to non-problem gamblers (Ladouceur and Walker 1996; Rogers 1998; Turner et al. 2006, 2008a). In addition, many harbor irrational beliefs, such as an illusion of control, a false notion that they can predict the outcome of a game, and a number of other erroneous beliefs about the games and their ability to beat the odds (Ladouceur and Walker 1996; Rogers 1998; Toneatto et al. 1997; Turner et al. 2008a; Vergura 2016; Wagenaar 1988). Such beliefs can lead to cognitive entrapment as they await the expected win (Rogers 1998). Furthermore, chasing after strategies based on these expected wins (e.g., doubling after a loss) can lead to further losses (Turner 1998; Turner and Horbay 2003). To deal with this problem, some interventions have been designed to educate people about how gambling actually works (e.g., the probabilities, the house edge, and the nature of random chance). A number of studies on prevention programs for problem gambling have focused on youth gambling (Turner et al. 2008b, c, d; Derevensky et al. 2007; Lavoie and Ladouceur 2004). In a study by Williams et al. (2010), results showed that students in an intervention group exposed to educational material had significantly more negative attitudes about gambling, improved resistance to gambling fallacies, improved decision-making and problem solving skills, as well as decreased gambling frequency and problems. Gallagher et al. (2011) reported that messages on EGMs about randomness led to reductions in time spent gambling. A study by Wohl et al. (2010) on adult gamblers found that when compared to participants who watched the control video, those who watched an animation that endorsed strategies to gamble within financial limits reported greater behavioral intentions to use the strategies and less often exceeded their pre-set limits during a subsequent gambling session.

On the other hand, some studies suggest that information-based interventions about the odds of winning are not effective. Williams and Connolly (2006) report on a study that found that math knowledge did not result in any change in gambling behavior in a college student population. A study by Monaghan and Blaszczynski (2010) found that pop-up messages related to self-appraisal were more effective than information-based messages.

These studies suggest that knowledge-based educational interventions have the potential to reduce the harm of gambling. To summarize, (1) it is known that many problem gamblers have a very poor understanding of random chance; (2) it is possible to teach people about random chance.

The intervention developed in this project takes a different approach. Rather than trying to teach problem gamblers about the probability of winning, the tutorial is designed to focus on the short- and long-term experience of gambling, how EGMs work, particularly how the game is designed to fool the players into believing they can win.

## Interactive Chasing and Volatility Demo

This paper describes the development and preliminary evaluation of an interactive EGM tutorial intended to help educate people on the design of video slot machine types of electronic gambling machines. We had two main groups in mind in designing this tutorial: problem gamblers in treatment and counselors who work with the clients. It was decided that counselors will be part of the study as they will likely need to provide assistance to the clients through the tutorial, and therefore would need to understand it themselves.

The primary purpose of the interactive tutorial is to educate gamblers and counselors about the nature of volatility and the futility of chasing related to EGMs. The set of topics selected for this tutorial was based on research into how EGMs work (e.g., Harrigan 2007; Harrigan and Dixon 2009; Jensen et al. 2013, Turner 2011a, b; Turner and Horbay 2004; Turner and Shi 2015). The tutorial is comprised of four modules designed to deal with several related aspects of EGMs, including volatility, the unpredictability of random chance, the continuously running nature of the RNG, prizes that are less than the amount bet, and the cost of play.

**Fig. 1:**
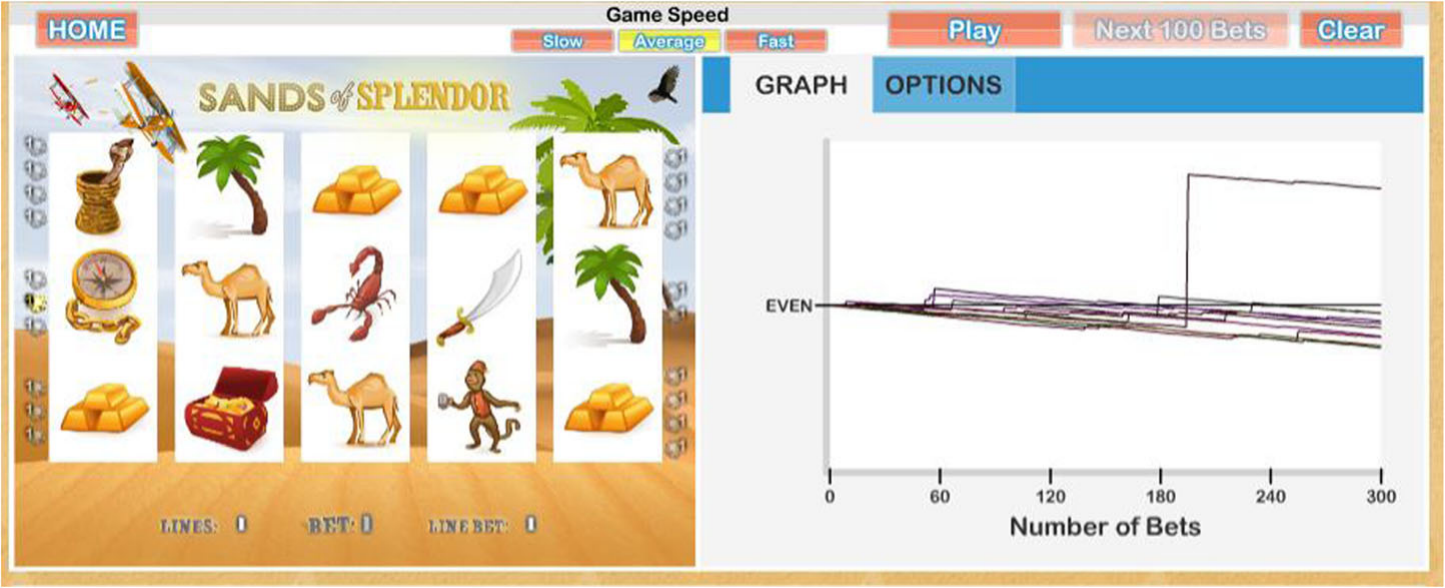
Screen shot of a draft version of the volatility demo illustrating the short-term outcome (a half hour of play)

**Fig. 2:**
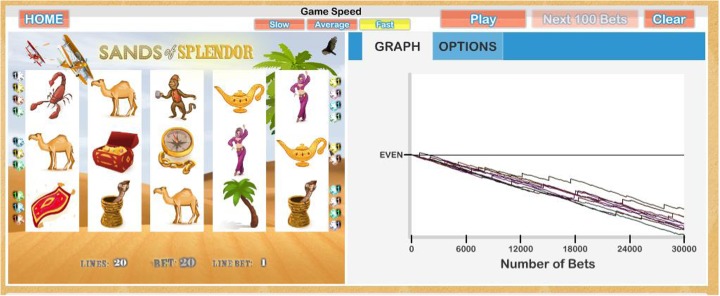
Screen shot of a draft version of the volatility demo illustrating the long-term outcome (50 h of play)

The first module deals with game volatility and the difference between short- and long-term experiences. Volatility is a measure of the variation in potential outcome from bet to bet. The gambling industry pays a lot of attention to game volatility when designing EGMs. Volatility is computed using the 90% confidence interval (*z* = 1.65) of the theoretical standard deviation of the outcome of a bet after 10,000 spins (see Harrigan and Dixon 2009; Kilby et al. 2004). Turner (2011b) argued that because of volatility, in the short term, it is difficult for a player to appreciate the long-term outcome. This is because in the short term, players win enough to give them the illusion that winning is possible, indeed even probable. However, in the long term, the games are designed to ensure that the casino earns a profit; the gambling industry does not like to gamble. In the tutorial, the user can simulate both short- and long-term gambling outcomes, which are illustrated with graphs. The player can run a single simulation or multiple simulations for a variety of bet sizes. They can also test their ability to predict the outcome over the next 100 spins. A screenshot of one component of the tutorial is shown in Figs. 1 and 2 to illustrate short-term and long-term outcomes (respectively) on an EGM.

A second module reveals the relative number of wins, losses, and false wins (when the prize is less than the amount bet). False wins are a consequence of having several pay lines each with its own multi-level prize structure. If the player covers several lines, small prizes on any particular line may be less than the total amount bet. Such wins are announced with the same fanfare sounds and lights as other wins, and thus provide reinforcement for continued gambling, even though the player has actually lost (Dixon et al. 2010; Jensen et al. 2013). The number of wins, losses, and false wins are displayed graphically.

The third module addresses one central aspect of chasing—the belief that one is due to win (Toneatto et al. 1997; Turner and Horbay 2004). Chasing is a central feature of problem gambling. Many players become cognitively entrapped by the game (Rogers 1998) and fear that if they walk away, someone else will “steal” their jackpot. To counteract this belief, we have also included in the demonstration a module that shows how the RNG runs continually. The users are invited to try and predict the outcome. This module also illustrates how the stop button provides no advantage to the player.

The final module allows the player to compute cost of play. The module allows players to input their typical pattern of play such as hours per day and days per year as well as their stakes (from 1 cent to $10), lines covered, and their speed of play. The tutorial then computes the total expected cost per session, per month, and per year. This can be an eye opener because few gamblers actually keep records of their spending. The client can be encouraged to play around with the options and find a level of loss that they are comfortable with.

To evaluate the interactive tutorial, it was presented to problem gamblers and gambling treatment counselors. The evaluation included a focus group with counselors, group presentations to clients, and counselors, and finally, individual sessions with both clients and counselors. In each case, the tutorial was provided to the participants online. Pen and paper questionnaires about random events were administered before and after viewing the tutorial. An additional 10-item evaluation questionnaire was administered after the tutorial.

The authors wish to emphasize that more knowledge is needed in the evaluation of the significance of cognitive-based interventions. The tutorial presented in this study must be viewed as a part of larger and more comprehensive efforts to reduce gambling-related harms.

## Hypotheses

1. Clients and counselors will provide high scores on the evaluation feedback questionnaire regarding the interactive chasing and volatility demo’s usability.
2. Clients and counselors will show a significant improvement in their understanding of EGMs after viewing the tutorial.
3. Counselors will score substantially higher than clients on the pre-test questionnaires.
4. Individual sessions will show more learning than group sessions.

## Methodology

The project was reviewed by the ethics review board of the (REMOVED FOR ANONYMITY) and approved as Protocol #055/2012. All procedures performed in studies involving human participants were in accordance with the ethical standards of the institutional and/or national research committee, as well as with the 1964 Helsinki declaration and its later amendments including informed consent and confidentiality of all personal information.

### Participants

In total, 52 participants completed the pre- and post-test questionnaire as well as the evaluation questionnaires: 26 clients (55% males) and 25 counselors (40% males). In addition, 16 counselors completed the evaluation questionnaire only. Sessions were either run as group educational sessions (clients *N* = 16; counselors *N* = 15) or individual sessions (clients *N* = 10; counselors *N* = 11). All individual sessions occurred in Toronto. The client (*n* = 16) and one of the counselor group (*N* = 5) sessions occurred at a retreatment site in West Virginia. Another counselor group was run in Toronto (*N* = 10). The first author ran all group sessions, PF, FJ, and NT each ran some of the individual sessions. Data from the two counselor group sessions were combined.

### Procedure

The evaluation of the tutorial was established using six different samples as described in the previous section. Two different methods were used in the study. Samples 1, 2, 3, and 4 were run as a group where the experimenter controlled the computer and demonstrated the various aspects of the program to the group. The sessions were run interactively, which means that the audience was asked to provide input whenever possible, such as which options to explore or what bets to examine. The sessions began with a consent form that was read and signed. For samples 2, 3, and 4, the researcher then handed out questionnaires. The participants were told to keep their questionnaires until the end, but hand in their consent forms so that the data would be anonymous. Then the researcher demonstrated each of the four modules of the program. After going through all of the modules, the researchers handed out the follow-up questionnaire and the evaluation form. The pre- and post-questionnaires were then collected together. All sessions allowed for an interactive discussion of the material. The individual sessions were roughly the same as the group sessions, except that the participant sat in front of the computer and controlled the mouse. The researcher sat beside the participant and gave instructions to try the various options of the program. The script used to guide the participant through the program is available from the first author.

### Questionnaires

The questionnaires contained no identification or confidential information.

Because of the setting, we needed to keep the evaluation and the pre- and post-tests short. The pre- and post-tests consisted of two questionnaires before and after the tutorial and an evaluation questionnaire after the tutorial:

1. A shortened version of the Random Events Knowledge Test (REKT; Turner et al. 2006). The REKT is a test of how well people understand the concept of random chance (alpha = .70). However, due to time limitations, we only included the 14 that were most relevant to EGMs in this tutorial.
2. A 9-item true-or-false content questionnaire was created specifically for this study that included questions that were directly addressed by the tutorial.
3. The 10-item evaluation questionnaire asked the participants if they agreed or disagreed with a series of statements about the tutorial such as “The software was easy to use.” Three of the items were reverse keyed to ensure that the person was reading each item. The counselor and client versions were slightly different with counselor version asking about “a client’s” responses, whereas the client version asked them about their own response.

### Data Analysis/Results

For hypothesis 1, we examined the descriptive statistics to determine how well the clients and counselors rated the quality of the demo program on the feedback questionnaire.

For hypothesis 2 (for both counselors and clients), the questionnaires were examined using pair *t* tests. In each case, it was predicted that there would be a significant difference between time 1 and 2 data.

For hypothesis 3, we used a *t* test to compare the pre-test scores of the counselors and clients.

For hypothesis 4, we compared the effect size for individual and group sessions.

## Results

### Evaluation Results

Both clients and counselors completed the same rating items, but the wording for counselors was slightly different.

As shown in Table 1, nearly all of the participants agreed or strongly agreed that the software was easy to use, easy to understand, and may be effective at preventing problem gambling. Reverse keyed items such as “This software is unlikely to stop anyone from problem gambling” produced mixed responses. In fact, 24% of the clients endorsed agree or strongly agree. However, 56% endorsed disagree or strongly disagree. This item was rated more positively by counselors; a strong majority (78%) of the counselors endorsed disagree or strongly disagree. The pattern of responses was similar for clients and counselors. However, three evaluation items showed significant differences between the clients and the counselors. The counselors were more skeptical than the clients about "The demo will help reduce my (the client’s) cravings to gamble"; *t*(64) = 2.8, *p* < .01. In addition, clients were more likely to disagree with the statement "While using the demo I wanted (clients may want) to gamble"; *t*(64) = 3.5, *p* < .001, and were somewhat more likely to endorse the item "The software was easy to understand"; *t*(62) = 1.9, *p* = .06.

**Table 1:** Client and Counselor evaluation data

Evaluation question		*N*						
			Strongly agree (%)	Agree (%)	Neither agree nor disagree (%)	Disagree (%)	Strongly disagree (%)	
The software was easy to use.	T	42	26	64	5	5	0	
	C	25	40	60	0	0	0	
The software was easy to understand.	T	42	21	67	12	0	0	*
	C	25	44	48	4	4	0	
The software program was very confusing.	T	42	0	0	10	76	14	
	C	25	4	4	4	56	32	
The software may be effective in preventing problem gambling.	T	42	10	74	14	2	0	
	C	25	32	48	16	0	4	
This software is unlikely to stop anyone from problem gambling.	T	42	2	7	12	76	2	
	C	25	8	16	20	40	16	
The software illustrated how much I can lose playing on a slot machine.	T	42	64	36	0	0	0	
	C	25	52	44	0	4	0	
The demo will help reduce my (the client’s) cravings to gamble.	T	41	5	32	49	15	0	**
	C	25	28	44	16	12	0	
While using the demo, I wanted (clients may want) to gamble.	T	41	5	20	46	24	5	**
	C	25	0	12	12	52	24	
Because of the demo, I now understand how slot machines work.	T	42	19	69	5	7	0	
	C	25	28	68	0	4	0	
The information about why chasing does not work will alter my gambling behavior (will impact the behavior of gamblers).	T	41	17	59	20	2	2	
	C	24	29	38	25	4	4	

*T* counselors, *C* clients. Where possible the same questions were used for both groups; however, for some items, the counselors responded to a slightly different version indicated in brackets. The asterisks in the last column indicates differences between groups: *< .05; **< .01

To test hypotheses 2, 3, and 4, questionnaires were administered before and after the tutorial: a short version of the REKT and a content test. The short REKT had a pre-test reliability of .76 and the content questionnaire had a retest reliability of .56.

For the content test, analysis of variance found a significant difference between pre-test and post-test scores overall, *F*(1, 47) = 17.6, *p* < .001 (pre-test < post-test). In addition, there was a significant main effect of participant group (client < counselor), *F*(1, 47) = 10.3, *p* < .001. Moreover, there was a significant interaction between test time with participant group, *F*(1, 47) = 6.5, *p* < .05. Finally, there was an interaction of participant group and presentation format *F*(1, 47) = 6.0, *p* < .05. An examination of this effect revealed that the clinicians who were tested in the individual sessions scored higher on both pre-test and post-test than the clinicians who participated in the group session; *t*(23) = 2.1, *p* < 05, whereas the clients who were in the group or individual session did not differ; *t*(24) = − 1.5, ns. There was no other main effect or interaction.

Table 2 gives the means of the content test for the counselors and clients and of the leaning effect for each group. The overall difference between pre-test and post-test was significant with an effect size of *d* = − .54. Examination of the means showed significant improvement for both counselors and clients with a somewhat stronger effect for clients (*d* = − .67) than for counselors (*d* = − .45).

**Table 2:** Pre- and post-content test scores across samples

	*N*	Pre-test		Post-test		Mean Diff	SD diff	*D*	*P*
		Mean	SD	Mean	SD				
Group counselors	14	7.71	0.73	8.29	0.47	− 0.57	0.75	− 0.76	**
Group clients	16	7.44	1.06	8.25	0.56	− 0.81	1.07	− 0.76	**
Individual counselors	11	8.46	0.99	8.55	0.50	− 0.09	0.79	− 0.11	Ns
Individual clients	10	7.60	0.66	8.20	0.40	− 0.60	0.80	− 0.75	*
All counselors	25	8.04	0.92	8.40	0.49	− 0.36	0.79	− 0.45	*
All clients	26	7.04	1.63	8.27	0.59	− 1.23	1.83	− 0.67	**
Total	51	7.53	1.42	8.33	0.55	− 0.80	1.48	− 0.54	***

*ns* not-significant

*p < .05; **p < .01; ***p < .001

For the short REKT, the overall difference between pre-test and post-test was significant *F*(1, 48) = 4.0, *p* < .05 (one tail). There was a significant interaction between participant type and time *F*(1, 48) = 9.9, *p* < .01. No other main effect or interaction was significant. As shown in Table 3, the REKT only showed significant improvement for the clients, not for the counselors. The short REKT data for each sample group is given in Table 3. In marked contrast to the content test, the short REKT showed little overall improvement (*d* = − .25) as a result of the tutorial. The results with the short REKT showed significant overall improvement for the clients (*d* = − .54) but not for the counselors (*d* = .23), which explains the interaction between participant type and test session.

**Table 3:** Pre- and post-short REKT scores across samples

Sample	*N*	Pre-test		Post-test		Mean diff	SD diff	*D*	*P*
		Mean	SD	Mean	SD				
Group counselors	15	13.33	0.72	13.27	0.96	0.06	0.76	0.08	ns
Group clients	16	10.50	1.80	11.25	1.30	− 0.75	1.75	− 0.43	ns
Individual counselors	11	13.73	0.45	13.27	1.14	0.46	0.89	0.48	ns
Individual clients	10	9.90	3.11	11.50	2.50	− 1.60	2.25	− 0.71	+
All counselors	25	13.52	0.64	13.28	1.04	0.24	1.03	0.23	
All clients	26	10.27	2.41	11.35	1.86	− 1.08	2.00	− 0.54	*
Total	52	11.89	2.39	12.31	1.78	− 0.42	1.71	− 0.25	ns

*ns* not-significant

+*p* = .05; *p < .05; ***p < .001

For hypothesis 3, that counselors will score substantially higher than clients on the pre-test questionnaires, we examined the difference between client and counselor scores. The content test was significantly higher for counselors at the pre-test, *t*(49) = 2.7, *p* < .05, *d* = .37, but was not significantly different at post-test, *t*(49) = 0.9, ns*, d = .14*. The short REKT scores were significantly higher for the counselors than for clients for both the pre-test, *t*(50) = 6.5, *p* < .001, *d* = .92, and the post-test *t*(50) = 4.6, *p* < .001, *d* = .64, but the difference had decreased.

The fourth hypothesis that individual sessions would produce more learning was not supported with either the content test or the short REKT; there was no main effect of group or interaction involving group.

We also looked at the specific items to identify the largest improvements in knowledge. As shown in Tables 4 and 5, the items that showed the largest changes were those most closely related to the content of the tutorial. For both the content test (Table 4) and the short REKT (Table 5), counselors scored perfect on several items on both the pre-test and post-test. For the clients, all nine content items showed some improvement, but items 1, 2, 5, 7, and 9 showed the largest effects for clients and for the counselors; items 1 and 6 showed the largest effects. Similarly, as shown in Table 5, clients showed the most improvement on items 2, 5, 13, and 14 on the short REKT items. Items 2, 5, and 14 each are specifically about EGMs, and item 13, although about lottery tickets, is structurally similar to item 14.

**Table 4:** Effect size of each content test items

	Clients (*n* = 26)				Counselors (*n* = 25)			
	*M*	SD	*D*		*M*	SD	*D*	
1. The random number generator runs continuously and the values are always changing even if no one is playing. (T)	− 0.23	0.43	− 0.54	*	− 0.16	0.37	− 0.43	*
2. It is difficult to appreciate the house edge because the losses are hidden by the occasional win. (T)	− 0.23	0.51	− 0.45	*				
3. The longer one plays, the more one loses. (T)	− 0.08	0.27	− 0.28					
4. If I keep playing, I will likely win back what I have lost. (F)	− 0.08	0.28	− 0.29					
5. In the long term, a player loses because of the house edge. (T)	− 0.12	0.33	− 0.35		− 0.04	0.35	− 0.11	
6. The house edge comes from the fact that the game does not pay out enough for wins to make up for the times the player loses. (T)	− 0.08	0.27	− 0.28		− 0.12	0.33	− 0.36	+
7. The house edge means that the game is not truly random. (F)	− 0.23	0.65	− 0.35	+	0.04	0.61	0.07	
8. Many of the wins on a multiline slot machine are smaller than the total amount that the player actually bets. (T)	− 0.04	0.45	− 0.09					
9. A loss is just a step toward a win. (F)	− 0.12	0.33	− 0.35	+	− 0.04	0.20	− 0.20	

There were no changes for counselors on items 2, 3, 4, and 8 because the counselors scored perfect on those items. The correct answer is indicated after each item with a T or an F

**Table 5:** Effect size of each short REKT test item

	Clients (*n* = 26)				Counselors (*n* = 25)		
	*M*	SD	*D*		*M*	SD	*D*
1. Knowledge of math can help you to win at lotteries. (F)	− 0.04	0.34	− 0.11		0.08	0.39	0.20
2. Staying at the same slot machines improves your chances of winning. (F)	− 0.27	0.45	− 0.60	**	− 0.04	0.20	− 0.20
3. Betting the same numbers for every lottery draw will not help you win. (T)	0.00	0.63	0.00		0.12	0.43	0.27
4. If you lose several times in a row, you are most likely to win if you keep playing. (F)	− 0.15	0.54	− 0.28				
5. Looking for a machine that has not paid out in a while will help you win. (F)	− 0.27	0.45	− 0.60	**	0.00	0.28	0.00
6. If you win three times in a row while gambling, you are less likely to win again, if you keep playing. (F)	0.00	0.40	0.00		0.04	0.34	0.11
7. Even by studying past winning numbers in a lottery, you cannot predict the winning numbers. (T)	0.00	0.50	0.00				
8. It would be foolish to bet on the number 18, if 18 had come up recently. (F)	0.08	0.56	0.14		0.04	0.20	0.20
9. If you flip a coin 5 times and you get heads 5 times in a row, you are most likely to get tails if you flip the coin again. (F)	− 0.04	0.45	− 0.09		− 0.08	0.27	− 0.28
10. If you have lost at several games in a row, your likelihood of winning or losing does not change. (T)	0.00	0.49	0.00		0.04	0.20	0.20
11. In a lottery, all numbers have the same chance of winning. (T)	0.04	0.20	0.20		0.00	0.28	0.00
12. If you always bet with the same numbers, are you more likely to win, no difference, less likely to win? (No difference)	− 0.08	0.27	− 0.28				
13. If you choose a random looking ticket number like 4692, are you more likely to win, no difference, or less likely to win than if you choose a non-random looking ticket number like 1234. (No difference)	− 0.12	0.33	− 0.35				
14. Suppose you are playing the slot machines and you’ve just won three times in a row. If you played again, do you think you would be more likely to win than usual, no difference, or less likely to win than usual. (No difference)	− 0.19	0.40	− 0.48	*	0.04	0.34	0.11

There were no changes for counselors on items 4, 7, 12, and 13 because the counselors scored perfect on those items. The correct answer is indicated after each item with a T or an F

## Discussion

The purpose of this study was to evaluate the impact of an educational online tutorial designed to teach people about the nature of electronic gambling machines or EGMs. The tutorial consists of four modules that expose a number of facts about EGMs, including (1) how short-term volatility obscures the long-term results, (2) the continuously running nature of the RNG, (3) the cost of playing, and (4) the number of false wins that a player would encounter.

The tutorial was evaluated using two methods: an evaluation questionnaire and a pre-test/post-test comparison of knowledge. Clients and problem gambling counselors were asked to rate the tutorial with 10 items including questions about how “easy” it is to understand, if it was “confusing”, or if it “may be effective in preventing problem gambling” on a 5-point agreement scale. The results show that both the clients and counselors rated the tutorial very positively. Notably, most of the clients and counselors agreed that the tutorial was easy to use and did not feel it was confusing. Overall, the clients were somewhat more enthusiastic about the tutorial than the counselors.

In addition, the tutorial was evaluated using two pre-test/post-test questionnaires: a content questionnaire and a short version of the REKT (Turner et al. 2006). Confirming hypothesis 2, both clients and counselors showed a significant increase in their scores on the content questionnaire. However, only the clients showed improvement on the short REKT. The counselors in the group administration condition did show some learning on the content questionnaire. The counselors in this group were new counselors who had not received specific training on EGMs, indicating that the tutorial can be effective in teaching new counselors on how EGMs work.

Confirming hypothesis 3, for both the content test results and the short REKT, the clients scored substantially lower at the pre-test compared to the counselors. In the post-test for the content questionnaire, the clients were not significantly different from the counselors indicating that the tutorial moved the clients from less knowledge of EGMs than the counselors to a level of knowledge of the EGMs roughly equal to that of the counselors. This indicates that the change in scores on the content question among the clients was a clinically significant improvement in knowledge (c.f., Jacobson and Truax 1991). For the short REKT, the post-scores were still significantly higher for the counselors than for the clients, but the difference at post-test was smaller. The effect size difference between clients and counselors shrunk from a very large effect of *d* = .92 to a moderate effect size of *d* = .62. The failure to show any learning for the counselors on the short REKT is not surprising because the pre-test scores on the short REKT were rather high with a pre-test score of 13.5 (*SD* = .65) out of 14, and thus there was not much room for improvement on the short REKT. Even on the content test, the counselors showed high pre-test scores of 8.00 (*SD* = .91) out of 9.

Overall, the counselors had perfect scores on four items on the short REKT and four items on the content test. It would appear that the current education and training system does a very good job of teaching counselors about random events; however, some improvement can be made in their understanding of EGMs.

An item-by-item examination revealed that some of the items produced substantial improvements in knowledge while others showed little or no evidence of improvement. It is particularly gratifying that item 2 of the content test (e.g., “It’s difficult to appreciate the house edge because the losses are hidden by the occasional win”), showed significant improvement with the clients because this was the concept that originally lead to the tutorial. Namely, that game volatility makes it difficult to appreciate the house edge (e.g., Turner 2011b). On the whole, the largest improvements were found for items that were most directly related to the content of the tutorial. The lack of any learning on the other items suggests that the tutorial lessons did not generalize to these items. The results suggest that the knowledge taught by the tutorial does not generalize to other random event situations that are based on the same principles. For example, item 5 of the short REKT showed significant improvement from pre-test to post-test, but item 7 showed none even though the principle behind the item is essentially the same.

This short evaluation study only examined the immediate impact of the tutorial. The tutorial is still under development and already has added features. Studies by Gallagher et al. (2011) and Wohl et al. (2010) have shown that it is possible in change gambling behavior by educating people about gambling. These studies suggest that knowledge-based educational interventions have the potential to reduce the harm of gambling. The current study offers another tool that can be used to educate counselors and clients about EGMs. Further study is needed to determine if the learning effect is sustained over time.

### Limitations

The samples were not random, but samples of volunteers. This means that caution must be exercised in generalizing the results. However, because the pre- and post-test data come from a within-subject experimental manipulation, we can conclude that the tutorial was effective at improving the knowledge of EGMs for clients and for some of the counselors. Another limitation is that the knowledge was evaluated immediately after the tutorial. It is unknown whether there was any long-term effect. Furthermore, questions concerning the effects of cognitive enhancement on actual behavior remain unanswered. Finally, we do not know if this tutorial can be used as a self-help tool. Currently, in order to get the most out of the tutorial, the problem gambler has to be guided through the tool which makes groups sessions more efficient.

## Conclusion

In summary, we found that the interactive tutorial evaluated in this study was positively evaluated by both clients and counselors and produced a significant improvement in scores on a content test about EGM games. This tutorial can be used (1) to educate counselors or gamblers about how EGMs work and (2) as a CBT treatment tool for sessions with clients. In the future, we will test the possibility of this tool being used as an online self-directed psycho-educational tool for problem gamblers or anyone in the general public. At the time of publication, a revised tutorial is available for free and can be accessed through the Internet. Contact the first author for information about the tutorial or for a copy of the script used to guide people through this program. The script can be refined into lesson plans for more general use of the tutorial for counselors and clients. This initiative must be viewed as a subset of more comprehensive efforts to reduce the harms related to disordered gambling. The question of cognition—and its place in our overall efforts to curb disordered gambling—remains unanswered.
